# A Successful Root Implantation

**Published:** 1913-04-15

**Authors:** Vincenzo Guerini

**Affiliations:** Italy


### A SUCCESSFUL ROOT IMPLANTATION.

BY DR. VINCENZO GUERINI (ITALY).

During the month of April of the present year, a Swedish painter, Carlo N., aged 27, residing at Positano (Naples) consulted me. His left cheek was swollen, the zygomatic region red, and the conjunctiva of the eye injected with blood, the left maxillary region painful to touch, with continuous pain over the cheek extending to the eye.

I examined the oral cavity, and noticed a fistulous opening on the gum over the left upper lateral incisor, the crown of which was absent, and the root had had its canal stopped with gutta-percha. Interrogating the patient he told me that more than two years back he had consulted a dentist in Sweden because of caries in the upper lateral incisor. The tooth was stopped after lengthy treatment; but pain set in, and a fistula showed itself on the gum. The stopping was then removed, and the medication of the tooth resumed until such time as the patient left Sweden for Paris, where he placed himself under the treatment of a French dentist, who continued medication for some time, and then thought well to cut off the crown and utilize the root for an artificial crown or pivot tooth.

But the patient got worse again, the swelling returned with severe pains in the face; so much so, that the dentist removed the artificial crown and substituted another pivot tooth with a metal tube for permanent drainage.

The patient having left Paris for Rome, and the pain and swelling increasing, consulted an expert specialist in the latter city, who rightly considered that the first thing was to remove this undesirable artificial crown. He treated the root for some time, and finally stopped the canal with gutta-percha. The patient then left Rome, and returned to his work at Positano. There, however, notwithstanding all previous treatment the patient continued to suffer. The cheek became greatly swollen and inflamed, the gingival fistula continually emitting pus in abundance with acute pain, allowing the patient no rest by day or by night. He now came to me in Naples presenting the symptoms previously described. I advised that the diseased root should be immediately extracted. This was found to be necrotic, the apex black and absorbed. Probing of the alveolus showed that it was pierced with an extension into a pathological cavity.

For the purpose of scraping this cavity, I introduced a small, sharp spoon, and in addition applied it to the apex of the root of the central incisor, as the affection had extended to that. This state of things caused me to reflect that although the offending root had been removed, there was still danger that infection might continue.

I therefore judged it necessary to drill through the cingulum of that tooth and remove the dead pulp, dilate the canal, and after mechanical and chemical disinfection immediately stop the canal itself with cement, driving this in, even beyond the apical foramen. This done I removed the superfluity through the sufficiently large opening and, then proceeded to complete the scraping of the pathological cavity and its windings, scrupulously and minutely, alternating this operation with energetic spraying of tepid solution of corrosive sublimate lotion, to rid the cavity of detritus and necrotic shreds of tissue.

My patient was greatly disturbed at the idea of being obliged to wear a prosthetic apparatus for the rest of his life. Wishing to set his mind at rest, I reflected on the possibility of an implantation, and remembering that I had a right canine tooth, perfectly sound and aseptically kept, drawn from the mouth of a young lady a year before (on account of its having grown in an abnormal position), I removed its crown, and, after cleansing and disinfecting, implanted the root (with the root canal stopped with gutta-percha) in the alveolus of the extracted lateral. I applied it without ligaturing, setting it in the alveolus, and driving it in by gentle tapping. I finished the operation, which lasted altogether about two hours, by painting with tincture of iodine the operated and surrounding parts.

On the following day the patient's face was much less swollen and red; the pain had ceased, so much so that he had slept the whole night through; the gum was of a pale rose color, the fistulous opening had closed, and no trace of pus was to be seen.

Afterward I begged him to come back in two months, in order to leave time for the growth of the bony tissue that should surround the implanted root as well as the radicular apex of the central incisor. Two months after, when the patient returned, I applied to the implanted root a porcelain crown with platinum pin, with a lingual surface of fused gold, having the normal anatomical form.—*British Journal.*

